# Novel TSPO Ligand 2-Cl-MGV-1 Can Counteract Lipopolysaccharide Induced Inflammatory Response in Murine RAW264.7 Macrophage Cell Line and Lung Models

**DOI:** 10.3390/cells13201702

**Published:** 2024-10-15

**Authors:** Fadi Obeid, Meygal Kahana, Baraah Dahle, Sheelu Monga, Yaniv Zohar, Abraham Weizman, Moshe Gavish

**Affiliations:** 1 Ruth and Bruce Rappaport Faculty of Medicine, Technion—Israel Institute of Technology, Haifa 31096, Israel; fadi_o12@hotmail.com (F.O.); meygal49@gmail.com (M.K.); braahemran@gmail.com (B.D.); sheelumonga@hotmail.com (S.M.); yanivz@technion.ac.il (Y.Z.); 2 Institute of Pathology and Cytology, Rambam Health Care Campus, P.O. Box 9602, Haifa 3109601, Israel; 3 Laboratory of Biological and Molecular Psychiatry, Felsenstein Medical Research Center, Faculty of Medicine, Tel Aviv University, Tel Aviv 6997801, Israel; weizmana@gmail.com; 4 Research Unit, Geha Mental Health Center, Petah Tikva 4910002, Israel

**Keywords:** inflammatory response, cytokines, lipopolysaccharide (LPS), 2-Cl-MGV-1, translocator protein (TSPO), RAW264.7 macrophages

## Abstract

We assessed the anti-inflammatory activity of the TSPO ligand 2-Cl-MGV-1. Lipopolysaccharide (LPS) was used to induce inflammatory response in a murine RAW264.7 macrophage model (LPS: 100 ng/mL) and a mouse model (C57BL/6) of lung inflammation (LPS: 5 mg/kg). In the macrophage model, the presence of 2-Cl-MGV-1 (25 µM) caused the LPS-induced elevation in nitrite levels to decrease by 70% (*p* < 0.0001) and interleukin (IL)-6 by 50% (*p* < 0.05). In the mouse model, 2-Cl-MGV-1, administered 30 min before, or co-administered with, an LPS injection, significantly inhibited the elevation in serum IL-5 levels (both by 65%; *p* < 0.001 and *p* < 0.01, respectively). 2-Cl-MGV-1 administration to mice 30 min before LPS injection and 1 h thereafter significantly inhibited the elevation in IL-1β serum levels (both by 63%, *p* < 0.005). IL-6 elevation was inhibited by 73% (*p* < 0.005) when 2-Cl-MGV-1 was administered 30 min before LPS, by 60% (*p* < 0.05) when co-administered with LPS, and by 64% (*p* < 0.05) when administered 1 h after LPS. All cytokine assessments were conducted 6 h post LPS injection. Histological analyses showed decreased leukocyte adherence in the lung tissue of the ligand-treated mice. 2-Cl-MGV-1 administration 30 min prior to exposure to LPS inhibited inflammation-induced open field immobility. The beneficial effect of 2-Cl-MGV-1 suggests its potential as a therapeutic option for inflammatory diseases.

## 1. Introduction

Systemic inflammation (SI) is an exaggerated response of the immune system triggered by a broad spectrum of pathological conditions and infections [1]. While inflammation typically acts as a protective mechanism that aims to maintain homeostasis in the human body, severe and prolonged inflammatory responses may result in severe and irreversible organ damage, leading to multi-organ dysfunction [2,3]. One should also keep in mind that SI is a significant contributor to the pathogenesis of life-threatening diseases such as sepsis, neuroinflammation, and neurodegenerative and autoimmune diseases [4,5,6].

While the precise mechanisms underlying SI are not yet fully understood, studies suggest that a complex set of stimuli triggers the activation of various immune blood cells, particularly lymphocytes, neutrophils, and macrophages [7]. This, in turn, leads to the over-secretion of several pro-inflammatory mediators, including cytokines, chemokines, and reactive oxygen species [8]. The cytokines released during this process include interleukin (IL)-6, IL-5, and IL-1β.

IL-6 is a soluble cytokine with a pleiotropic effect on inflammation, immune response, and hematopoiesis [9,10]. It is synthesized in local lesions in the initial stage of inflammation and moves to the liver through the bloodstream. This process initiates the rapid synthesis of acute-phase proteins such as C-reactive protein (CRP), serum amyloid A (SAA), fibrinogen, haptoglobin, and alpha1-antichymotrypsin [11].

Other cytokines relevant to inflammation include IL-1β, also known as lymphocyte-activating factor [12], and IL-5, which is involved in the differentiation, recruitment, survival, and degranulation of eosinophils and is particularly relevant to bronchial asthma [13].

Several medications are used for treating SI, but their efficacy is impeded by various factors, including suboptimal pharmacokinetic properties, off-target effects, and insufficient selectivity for key inflammatory pathways. Established drugs, including nonsteroidal anti-inflammatory drugs (NSAIDs), corticosteroids, and immunosuppressants, have demonstrated some effectiveness in attenuating the inflammatory response. Experience, however, raises concerns regarding their safety, tolerability, and long-term use and limits their clinical utility [14,15].

The translocator protein (TSPO) is an 18 KDa protein known to play a key role in numerous essential biological processes [16,17]. Recent studies have shown that TSPO is involved in systemic inflammatory responses [18,19]. In these cases, it is highly expressed in distinct immune cells, especially in macrophage cells [20]. A neuro-inflammatory study has shown that, in microglial cells, the anti-inflammatory effect of the ligand may be achieved by the suppression of NF-κB protein expression [19]. Another possibility is that the stimulation of steroid synthesis contributes to the anti-inflammatory effect of TSPO ligands [21].

Given the significant morbidity and mortality associated with diseases characterized by SI [22], and the scarcity of drugs to treat these conditions [15], there is clearly an unmet need for the development of novel therapeutic strategies to effectively mitigate pathological consequences and improve patient outcomes. As a key player in SI, TSPO presents a promising opportunity for targeted therapeutic interventions. Our lab has, therefore, developed a novel TSPO ligand called 2-Cl-MGV-1 that was characterized extensively [23] and shown to possess anti-inflammatory effects in in vitro models [18,19].

The present study aims to explore the therapeutic potential of TSPO immuno-modulatory activity by treating SI in in vitro and in vivo models using LPS-induced inflammation in a RAW264.7 macrophage cell line and the lungs of C57BL/6 mouse models of SI.

## 2. Materials and Methods

### 2.1. The In Vitro Cellular Model of Inflammation

RAW264.7 macrophage cells (ATCC, Rockville, MD, USA) were cultured at 37 °C with 5% CO_2_ in 90% relative humidity. Cells were incubated in Dulbecco’s modified Eagle’s medium (DMEM) containing 4.5 g/L glucose and 2 mM L-glutamine and supplemented with 10% fetal bovine serum, 1% sodium pyruvate, penicillin (100 U/mL), and streptomycin (100 µg/mL).

#### 2.1.1. Viable Cell Counting with Trypan Blue Exclusion Dye Assay

Cells were counted using Neubauer slides, whereby viable cells do not take up trypan blue dye, while non-viable cells absorb the dye and appear blue.

The process started with cells being scraped off the plates and suspended in a fresh medium. Next, a 200 µL sample was collected for cell counting. Trypan blue (Sigma-Aldrich, Rehovot, Israel) at a final concentration of 0.05% was used to stain the cell sample. The medium and dye were mixed at a volume ratio of 1:1 and the stained cells were counted using a hemocytometer with an inverted microscope (Sigma-Aldrich-Merck, Jerusalem, Israel). All cells were found to be viable and were counted in the in vitro groups described later in Materials and Methods.

#### 2.1.2. LPS Stimulation of Macrophages

RAW264.7 cells were seeded in a 12-well plate for 24 h. Subsequently, the cells were exposed to 100 ng/mL LPS (Sigma-Aldrich, Rehovot, Israel). Twenty-four hours after LPS exposure, the cells were trypsinized and the media samples were collected for several assays. The serotype of the LPS was *Escherichia coli* O55:B5.

#### 2.1.3. Nitrite Assay as an Inflammatory Marker

Nitrite production was determined by mixing 100 µL of cell culture supernatant with Griess reagent at a ratio of 1:1. Sodium nitrate (0.1 M) was used to create the calibration curve. Absorbance at 540 nm was measured with the Spectrophotometer Infinite^®^ 200 PRO (Tecan Trading AG, Männedorf, Switzerland) after leaving the sample on a shaker for 15 min.

#### 2.1.4. Enzyme-Linked Immunosorbent Assay for Assessment of Cytokine Levels

The levels of cytokines were assessed using specific enzyme-linked immunosorbent assay (ELISA) kits. All the samples were diluted according to the manufacturer’s instructions and cytokine levels were compared between groups. All the reagents came ready-for-use in commercial kits and were stored according to the manufacturer’s (Abcam, Cambridge, UK) instructions. The kits included the following: Mouse IL-6 ELISA Kit (ab222503; Abcam, Cambridge, UK): 11.3 pg/mL sensitivity. This kit recognizes both native and recombinant mouse IL-6 protein, as well as rat IL-6 protein. Mouse IL-1β ELISA Kit (ab197742; Abcam, Cambridge, UK): 1 pg/mL sensitivity. No cross-reactivity was observed with Mouse IL-1α and Mouse IL-1 receptor1. Mouse IL-5 ELISA Kit (ab204523; Abcam, Cambridge, UK): 1.2 pg/mL sensitivity.

#### 2.1.5. Assessing the Effects of 2-Cl-MGV-1 on RAW264.7 Inflammatory Response to LPS

RAW264.7 murine macrophages were exposed to 100 ng/mL of LPS with or without the TSPO ligand 2-Cl-MGV-1 (25 μM). The cell culture supernatant was collected and aliquoted and the cell lysates were prepared using a lysis buffer as previously described [24]. Alterations in nitrite content and cytokines in the supernatant were assessed.

### 2.2. The In Vivo Mouse Model for Systemic Inflammation

Male C57BL/6 mice (6 weeks old, 20 ± 2 g), obtained from Envigo (Rehovot, Israel), were housed in a pathogen-free room under controlled temperature (22–23 °C), humidity (55% ± 15%), and lighting (12-hour light–dark cycles) and were given access to food and water ad libitum.

The study was approved by the Technion committee for experiments in animals (IL-084-07-2020).

The mice were divided into five treatment groups:

- Vehicle: vehicle (20 µL DMSO; S.C.);
- LPS + vehicle: LPS (5 mg/kg; I.P.) + vehicle (20 µL DMSO; S.C.);
- Pre-administration group: 2-Cl-MGV-1 (7.5 mg/kg; S.C.) administered 30 min before LPS (5 mg/kg; I.P);
- Co-administration group: co-administration of 2-Cl-MGV-1 (7.5 mg/kg; S.C.) + LPS (5 mg/kg; I.P.) administered at the same time;
- Post-administration group [LPS+2-Cl-MGV-1(post LPS)]: 2-Cl-MGV-1 (7.5 mg/kg) S.C. administered 1 h after LPS (5 mg/kg) I.P. This group did not participate in the in vivo IL-5 experiment due to technical problems.

The mouse survival rate was 100%.

#### 2.2.1. Blood Collection

Blood was collected 6 h after LPS or vehicle administration. The mice were sacrificed by placing them in a closed container and introducing isoflurane via a vaporizer (5% isoflurane). They inhaled the vapors until respiration ceased and death ensued. They were then decapitated and blood samples were collected for cytokine assessments.

After 20 min at room temperature, the sera were separated by centrifugation and were stored at −80 °C until they were assayed for cytokines. Serum cytokine levels were determined with ELISA using a Spectrophotometer Infinite^®^ 200 PRO microplate reader (Tecan Trading AG, Männedorf, Switzerland).

#### 2.2.2. Histology of Lung Tissue

Mouse lungs were collected 6 h after LPS or vehicle administration and were then inflated with 3ml of neutral buffered formalin (4%) and fixed for 24 h [25]. Following fixation, lungs were processed using a Tissue-Tek VIP6 tissue processor (Sakura Finetek USA, Inc., Torrance, CA, USA) and were paraffin-embedded. Sections 4 µm in size were stained with hematoxylin–eosin and imaged at ×40 using a Nikon DS-Fi2 camera (Nikon Corporation, Tokyo, Japan). Analysis of adherent leukocyte density was performed using Image-Pro Plus v. 6.0 (Media Cybernetics, Rockville, MD, USA).

Five representative images of large pulmonary vessels were analyzed for each group. The inner circumference of each blood vessel was measured in pixels, and then adherent leukocytes were counted manually. The density of leukocyte adherence to endothelial cells was calculated as the number of leukocytes per 1000 pixels of blood vessel circumference. The adherence of leukocytes to the blood vessel walls represents an immediate inflammatory response.

#### 2.2.3. In Vivo Behavioral Assessment

Six hours after the administration of the various treatments, the mice were observed for 5 min in an open field (52.5 × 48.5 × 12  cm arena). For each mouse, it was recorded whether the mouse spent the whole 5 min staying in a corner (Corner).

### 2.3. Statistical Analyses

Results are presented as mean ± standard error of mean (SEM). The data were analyzed using the one-way analysis of variance (ANOVA) test followed by Bonferroni’s post hoc test. Fisher’s Exact Test was used for the categorical behavioral data. Statistical significance was set at *p* < 0.05.

For the in vitro cellular model of inflammation experiments, all samples were chosen to include 4 mice, as this size was deemed large enough for statistical analysis.

Initially, the samples for all cytokine experiments were chosen to include 4 mice for the vehicle group and 7 for the other groups. However, due to unknown and unexplained technical issues, no results were obtained in the IL-6 experiment for the “2-Cl-MGV-1 administered 1 h after LPS” group for one sample. The sample could therefore not be included in the analysis, so the size of this group in the experiment ended up being n = 6.

In the in vivo experiments, the groups containing vehicle were chosen to be larger (n = 10) than the other groups (n = 5, i.e., ratio of 2:1) in order to ascertain that the control groups were large enough to represent the normal range. For the behavioral experiments, we used Fisher’s Exact Test.

## 3. Results

### 3.1. In Vitro Cellular Model of Inflammation

**Figure 1:**
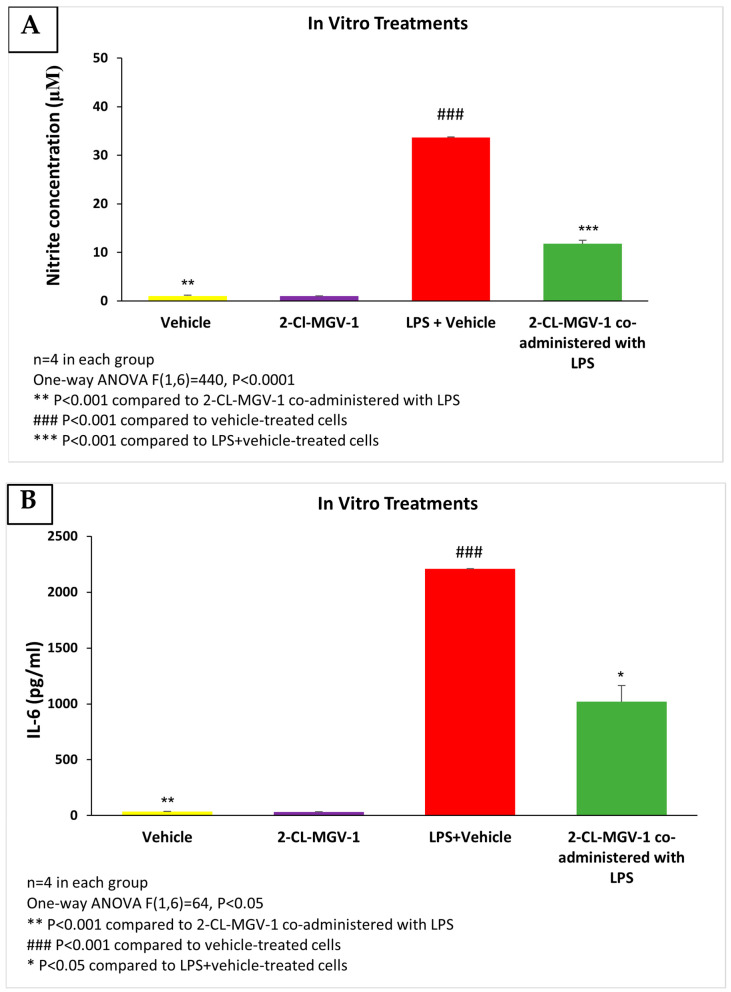
(**A**) Nitrite levels; (**B**) IL-6 levels in RAW264.7 cells exposed to 100 ng/mL of LPS and 25 µM of 2-Cl-MGV-1 for 24 h. ANOVA followed by the Bonferroni post hoc test was performed. Results were calculated using a standard calibration curve and are presented as mean ± SEM.

The exposure of RAW264.7 cells to LPS was associated with marked elevation in nitrite and IL-6 compared to vehicle (DMSO). These elevations were attenuated by 2-Cl-MGV-1 (Figure 1A,B). In this in vitro paradigm, 2-Cl-MGV-1 successfully inhibited the production of nitrite (65%, *p* < 0.0001) and reduced the release of the pro-inflammatory cytokine IL-6 (54%, *p* < 0.05) as compared to LPS (with or without vehicle). The ligand 2-Cl-MGV-1 alone did not affect the nitrite and IL-6 levels and they remained in the vehicle range.

### 3.2. In Vivo Mouse Model of Systemic Inflammation

Circulatory inflammatory cytokine levels increased considerably 6 h after LPS administration.

#### IL-6 Serum Levels

**Figure 2:**
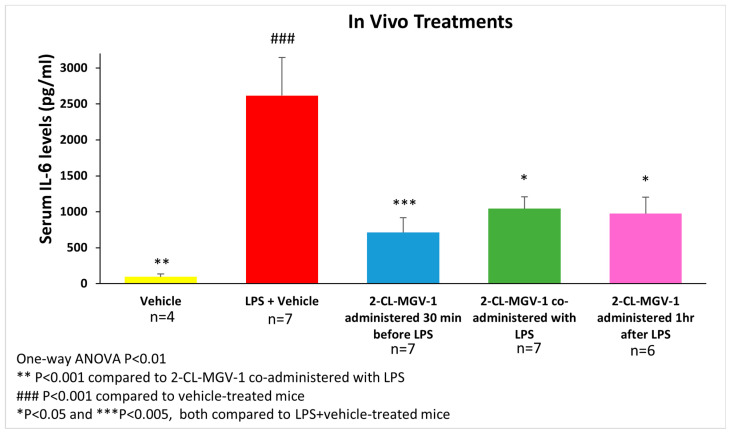
The impact of 2-Cl-MGV-1 administration on LPS-induced serum IL-6 elevation in C57BL/6 mice. Assessment of IL-6 serum levels was performed 6 h after administration of LPS (LPS—5 mg/kg I.P., 2-Cl-MGV-1–7.5 mg/kg S.C., vehicle—DMSO). Serum IL-6 levels (pg/mL) were assessed using a standard calibration curve and are presented as mean ± SEM. One-way ANOVA followed by the Bonferroni post hoc test was performed.

The IL-6 serum levels of C57BL/6 mice were assessed 6 h after administering LPS I.P. (5 mg/kg). 2-Cl-MGV-1 (7.5 mg/kg) administered 30 min before LPS inhibited the LPS-induced increase in IL-6 by 73% (*p* < 0.005), while co-administration with LPS inhibited IL-6 elevation by 60% (*p* < 0.05), and administration 1 h after LPS inhibited IL-6 elevation by 64% (*p* < 0.05 for all; Figure 2).

**Figure 3:**
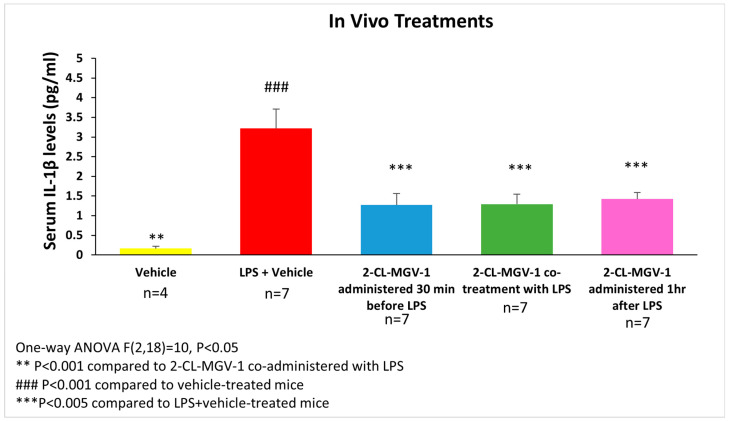
The impact of 2-Cl-MGV-1 administration on LPS-induced serum IL-1β elevation in C57BL/6 mice. Assessment of IL-1β serum levels was performed 6 h after administration of LPS (LPS—5 mg/kg I.P., 2-Cl-MGV-1—7.5 mg/kg S.C., vehicle—DMSO). IL-1β levels (pg/mL) were assessed using a standard calibration curve and are presented as mean ± SEM. One-way ANOVA followed by the Bonferroni post hoc test was performed.

Figure 3 shows the alterations in IL-1β serum levels following exposure to LPS. The IL-1β serum levels in the LPS+2-Cl-MGV-1 groups were about 56–61% lower than in the LPS+vehicle group. (*p* < 0.005 for all, Figure 3).

**Figure 4:**
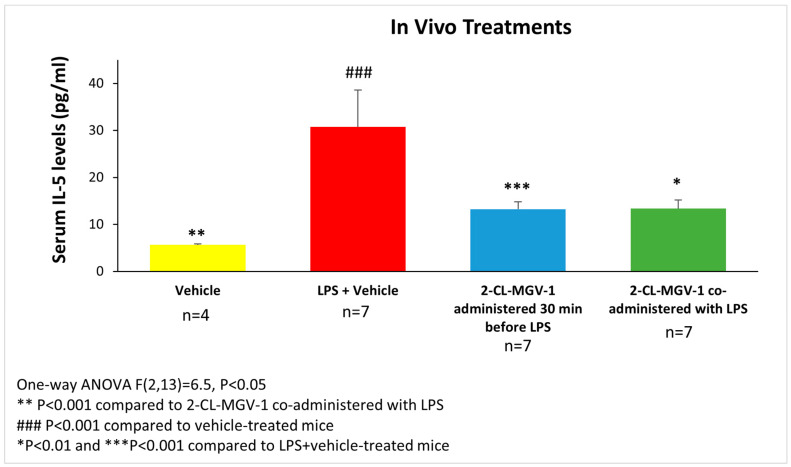
The impact of 2-Cl-MGV-1 administration on LPS-induced serum IL-5 elevation in C57BL/6 mice. Assessment of IL-5 serum levels was conducted 6 h after administration of LPS (LPS—5 mg/kg I.P., 2-Cl-MGV-1—7.5 mg/kg S.C., vehicle—DMSO). IL-5 levels (pg/mL) were measured using a standard calibration curve and are presented as mean ± SEM. One-way ANOVA followed by the Bonferroni post hoc test was performed.

Figure 4 shows the changes in IL-5 serum levels following the administration of LPS. The LPS+vehicle group showed a significant up-regulation of IL-5 serum levels. IL-5 serum levels in the LPS+2-Cl-MGV-1 groups when administered 30 min before, or co-administered with, LPS were significantly lower compared to the LPS+vehicle group (57% reduction for both, *p* < 0.001 and *p* < 0.01, respectively).

### 3.3. Histology of Lung Tissue

**Figure 5:**
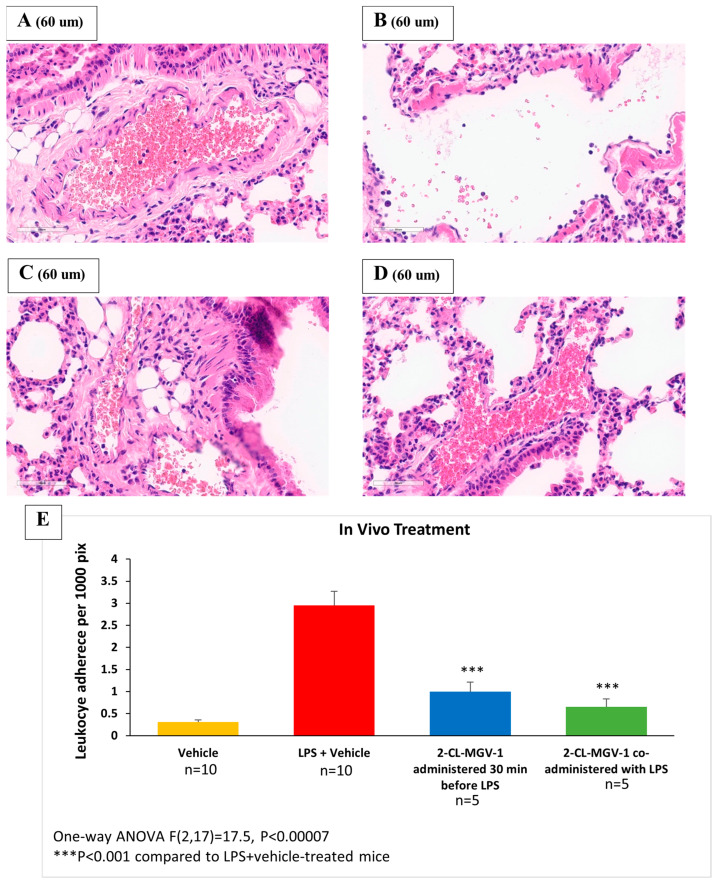
Representative histological slides of lung tissue. (**A**) Vehicle; (**B**) LPS+vehicle; (**C**) 2-Cl-MGV-1 administered 30 min before LPS; (**D**) 2-Cl-MGV-1 co-administered with LPS; (**E**) quantitative analysis of leukocyte adherence to endothelial cells in the pulmonary blood vessels (density per 1000 pixels) in the 4 groups. Histology of lung tissue was performed 6 h after LPS or vehicle administration. Results are expressed as mean ± SEM. ANOVA followed by the Bonferroni post hoc test was performed. Samples of adherent leukocytes are marked by circles.

As shown in Figure 5, LPS I.P. (5 mg/kg) administration was associated with a robust increase in leukocyte adherence to pulmonary blood vessels, while 2-Cl-MGV-1 (7.5 mg/kg S.C.) co-administration prevented the elevation of leukocyte adherence, leaving it in the vehicle range. The administration of 2-Cl-MGV-1 alone did not affect leukocyte adherence.

### 3.4. In Vivo Behavioral Assessment

Six hours after the administration of the various treatments, the elevation in IL-6 (Figure 2) was associated with depression-like behavior, reflected by staying in a corner of the cage for the whole 5 min of observation (Table 1). The administration of 2-Cl-MGV-1 30 min prior to the administration of LPS significantly inhibited the LPS-induced depression-like behavior. These results indicate the ability of the ligand to prevent the inflammation-induced suppression of mobility in an open field. No significant results were obtained, however, when the ligand was administered simultaneously with LPS or 1 h after LPS exposure (*p* = 0.07 for both; Table 1).

## 4. Discussion

The major finding of this study is that the TSPO ligand 2-Cl-MGV-1 exhibits anti-inflammatory activity in both in vitro cellular and in vivo LPS models of SI.

RAW264.7 macrophage cells and C57BL/6 male mice were used for the cellular and animal models of SI, respectively. It was shown that the novel TSPO ligand 2-Cl-MGV-1 inhibited LPS-induced inflammation, as reflected by the attenuation of nitrite accumulation and pro-inflammatory cytokine release (IL-6) in the medium (in vitro) and IL-1β, IL-6, and IL-5 in serum (in vivo). The immunomodulatory effect of 2-Cl-MGV-1 and other TSPO ligands has been previously reported in other cellular [18,26,27,28,29] and in vivo models of inflammation [30].

LPS IP administration to mice causes SI. The innovative TSPO ligand 2-Cl-MGV-1 attenuated the synthesis or the release of inflammatory molecules, or both. It is possible that the anti-inflammatory activity was achieved by the inhibition of NF-κB protein expression [19,31]. In a previous study, pre-treatment (3 days) of C57BL/6 mice with the classical TSPO ligand PK 11195, followed by 11 days of systemic chronic injection of LPS, attenuated LPS-induced cognitive dysfunction [25,31]. This protective effect was related to the normalization of cyclooxygenase-2 levels, increased synthesis of the protective neurosteroids progesterone and allopregnanolone, and down-regulation of β-site APP-cleaving enzyme-1 [31]. Similar neuroprotective effects were shown with etifoxine. The PK 11195 TSPO ligand also attenuated systemic LPS-induced neuroinflammation and cognitive dysfunction in C57BL/6 mice. Such anti-neuro-inflammatory activity was achieved by the alleviation of hippocampal inflammation, associated with an increase in brain neurosteroids [25,31].

In our mouse model of pulmonary inflammation, 2-Cl-MGV-1 prevented the inflammatory response to LPS, as reflected by a lesser adherence of leukocytes to endothelial cells in pulmonary blood vessels [32]. Such adherence is a marker of pulmonary inflammation (i.e., a bacterial-pneumonia-like condition). The ligand restored leukocyte adherence to the vehicle range (Figure 5).

On the behavioral level, the LPS-induced depression-like behavior (i.e., staying in the corner) in mice was significantly attenuated when the ligand was administered 30 min prior to LPS, and the protective effect approached significance (*p* = 0.07) when the ligand was co-administered or administered 1 h after exposure to LPS. Thus, it appears that the ligand can prevent the LPS-induced mobility deficit related to SI. Future studies should assess the effect of the ligand on LPS-induced deficits in social behavior and the increase in anxiety and depressive-like behavior [9]. Such studies should include the assessment of NF-κB protein, neurosteroids, and the relevant inflammatory pathways.

### Limitations

Unfortunately, in the present study, we did not analyze specific M1 and M2 markers. However, both in vitro and in vivo experimental approaches consistently demonstrated the suppression and even prevention of inflammatory pathways following treatment with 2-Cl-MGV-1.

In a previous study [19], we demonstrated that 2-Cl-MGV-1 is a suppressor of NF-κB protein expression. Unfortunately, in the present study, we did not assess the NF-κB protein expression in RAW264.7 macrophages.

Another limitation of this study is the fact that the authors did not investigate the effects of 2-Cl-MGV-1 on the inflammatory responses in macrophages and mouse lungs caused by LPS through TSPO protein signaling. Another study to this effect using TSPO-knockout cells and mice is planned for the near future.

Notably, in this study, only the low-affinity (2.6 ± 1.0 nM) 2-Cl-MGV-1 TSPO ligand was used in all experiments. A comparison to high-affinity TSPO ligands is needed to substantiate the role of TSPO in the immunomodulatory effects of 2-Cl-MGV-1. Although speculative, it is possible that the safety of the low-affinity ligand is better than that of high-affinity ligands.

## 5. Conclusions

In conclusion, we showed that the novel TSPO ligand 2-Cl-MGV-1 is a potent inhibitor of inflammatory pathways in cellular and animal models of inflammation. The anti-inflammatory effects may be mediated by a suppressive effect of the ligand on NF-κB protein and steroid expression [19,31]. TSPO may thus serve as a novel target for the treatment of inflammatory diseases. Further investigation of this ligand in acute and chronic inflammatory conditions, including autoimmune diseases, is warranted.

## Figures and Tables

**Table 1:** Behavioral assessment in open field.

	Staying in the Corner Corner/Total (%)	Statistics (Fisher’s Exact Test): P of Comparisons vs. LPS + Vehicle
Vehicle	0/4 (0)	0.003
LPS + Vehicle	7/7 (100)	-
2-Cl-MGV-1 administered 30 min before LPS	1/7 (14)	0.005
2-Cl-MGV-1 co-administered with LPS	3/7 (42)	0.07
2-Cl-MGV-1 administered 1 h after LPS	3/6 (50)	0.07

## Data Availability

The data presented in this study are available on request from the corresponding author.

## References

[1] Giridharan V.V., Generoso J.S., Collodel A., Sayana P., Barichello T., Rezaei N., Yazdanpanah N. (2023). Chapter 9—The impact of systemic inflammation on neuroinflammation. Translational Neuroimmunology.

[2] Arroyo V., Angeli P., Moreau R., Jalan R., Clària J., Trebicka J., Fernández J., Gustot T., Caraceni P., Bernardi M. (2021). The systemic inflammation hypothesis: Towards a new paradigm of acute decompensation and multiorgan failure in cirrhosis. J. Hepatol..

[3] Sun X., Jones Z.B., Chen X.M., Zhou L., So K.F., Ren Y. (2016). Multiple organ dysfunction and systemic inflammation after spinal cord injury: A complex relationship. J. Neuroinflamm..

[4] Ebrahim G.J. (2011). Sepsis, septic shock and the systemic inflammatory response syndrome. J. Trop. Pediatr..

[5] Zhang W., Xiao D., Mao Q., Xia H. (2023). Role of neuroinflammation in neurodegeneration development. Signal Transduct. Target. Ther..

[6] Pohl D., Benseler S. (2013). Systemic inflammatory and autoimmune disorders. Handb. Clin. Neurol..

[7] Kong N., Chen G., Wang H., Li J., Yin S., Cao X., Wang T., Li X., Li Y., Zhang H. (2021). Blood leukocyte count as a systemic inflammatory biomarker associated with a more rapid spirometric decline in a large cohort of iron and steel industry workers. Respir. Res..

[8] Closa D., Folch-Puy E. (2004). Oxygen free radicals and the systemic inflammatory response. IUBMB Life.

[9] Biesmans S., Meert T.F., Bouwknecht J.A., Acton P.D., Davoodi N., De Haes P., Kuijlaars J., Langlois X., Matthews L.J., Ver Donck L. (2013). Systemic immune activation leads to neuroinflammation and sickness behavior in mice. Mediat. Inflamm..

[10] Tanaka T., Narazaki M., Kishimoto T. (2014). IL-6 in inflammation, immunity, and disease. Cold Spring Harb. Perspect. Biol..

[11] Jain S., Gautam V., Naseem S. (2011). Acute-phase proteins: As diagnostic tool. J. Pharm. Bioallied Sci..

[12] Ren K., Torres R. (2009). Role of interleukin-1beta during pain and inflammation. Brain Res. Rev..

[13] Pelaia C., Paoletti G., Puggioni F., Racca F., Pelaia G., Canonica G.W., Heffler E. (2019). Interleukin-5 in the Pathophysiology of Severe Asthma. Front. Physiol..

[14] Wirth T., Lafforgue P., Pham T. (2023). NSAID: Current limits to prescription. Jt. Bone Spine.

[15] Cascorbi I. (2017). Inflammation: Treatment Progress and Limitations. Clin. Pharmacol. Ther..

[16] Papadopoulos V., Fan J., Zirkin B. (2018). Translocator protein (18 kDa): An update on its function in steroidogenesis. J. Neuroendocrinol..

[17] Biswas L., Farhan F., Reilly J., Bartholomew C., Shu X. (2018). TSPO Ligands Promote Cholesterol Efflux and Suppress Oxidative Stress and Inflammation in Choroidal Endothelial Cells. Int. J. Mol. Sci..

[18] Azrad M., Zeineh N., Weizman A., Veenman L., Gavish M. (2019). The TSPO Ligands 2-Cl-MGV-1, MGV-1, and PK11195 Differentially Suppress the Inflammatory Response of BV-2 Microglial Cell to LPS. Int. J. Mol. Sci..

[19] Monga S., Nagler R., Amara R., Weizman A., Gavish M. (2019). Inhibitory Effects of the Two Novel TSPO Ligands 2-Cl-MGV-1 and MGV-1 on LPS-induced Microglial Activation. Cells.

[20] Vicente-Rodríguez M., Singh N., Turkheimer F., Peris-Yague A., Randall K., Veronese M., Simmons C., Karim Haji-Dheere A., Bordoloi J., Sander K. (2021). Resolving the cellular specificity of TSPO imaging in a rat model of peripherally-induced neuroinflammation. Brain Behav. Immun..

[21] Rupprecht R., Pradhan A.K., Kufner M., Brunner L.M., Nothdurfter C., Wein S., Schwarzbach J., Puig X., Rupprecht C., Rammes G. (2023). Neurosteroids and translocator protein 18 kDa (TSPO) in depression: Implications for synaptic plasticity, cognition, and treatment options. Eur. Arch. Psychiatry Clin. Neurosci..

[22] Proctor M.J., McMillan D.C., Horgan P.G., Fletcher C.D., Talwar D., Morrison D.S. (2015). Systemic inflammation predicts all-cause mortality: A glasgow inflammation outcome study. PLoS ONE.

[23] Vainshtein A., Veenman L., Shterenberg A., Singh S., Masarwa A., Dutta B., Island B., Tsoglin E., Levin E., Leschiner S. (2015). Quinazoline-based tricyclic compounds that regulate programmed cell death, induce neuronal differentiation, and are curative in animal models for excitotoxicity and hereditary brain disease. Cell Death Discov..

[24] Monga S., Denora N., Laquintana V., Franco M., Marek I., Singh S., Nagler R., Weizman A., Gavish M. (2020). The protective effect of the TSPO ligands 2,4-Di-Cl-MGV-1, CB86, and CB204 against LPS-induced M1 pro-inflammatory activation of microglia. Brain Behav. Immun. Health.

[25] Zhang H., Ma L., Guo W.Z., Jiao L.B., Zhao H.Y., Ma Y.Q., Hao X.M. (2020). TSPO ligand etifoxine attenuates LPS-induced cognitive dysfunction in mice. Brain Res. Bull..

[26] Horiguchi Y., Ohta N., Yamamoto S., Koide M., Fujino Y. (2019). Midazolam suppresses the lipopolysaccharide-stimulated immune responses of human macrophages via translocator protein signaling. Int. Immunopharmacol..

[27] Bessler H., Caspi B., Gavish M., Rehavi M., Hart J., Weizman R. (1997). Peripheral-type benzodiazepine receptor ligands modulate human natural killer cell activity. Int. J. Immunopharmacol..

[28] Bessler H., Weizman R., Gavish M., Notti I., Djaldetti M. (1992). Immunomodulatory effect of peripheral benzodiazepine receptor ligands on human mononuclear cells. J. Neuroimmunol..

[29] Chen D.L., Agapov E., Wu K., Engle J.T., Solingapuram Sai K.K., Arentson E., Spayd K.J., Moreland K.T., Toth K., Byers D.E. (2021). Selective Imaging of Lung Macrophages Using [(11)C]PBR28-Based Positron Emission Tomography. Mol. Imaging Biol..

[30] Hatori A., Yui J., Yamasaki T., Xie L., Kumata K., Fujinaga M., Yoshida Y., Ogawa M., Nengaki N., Kawamura K. (2012). PET imaging of lung inflammation with [18F]FEDAC, a radioligand for translocator protein (18 kDa). PLoS ONE.

[31] Ma L., Zhang H., Liu N., Wang P.Q., Guo W.Z., Fu Q., Jiao L.B., Ma Y.Q., Mi W.D. (2016). TSPO ligand PK11195 alleviates neuroinflammation and beta-amyloid generation induced by systemic LPS administration. Brain Res. Bull..

[32] Davenport M.L., Sherrill T.P., Blackwell T.S., Edmonds M.D. (2020). Perfusion and Inflation of the Mouse Lung for Tumor Histology. J. Vis. Exp..

